# Anterograde Intramedullary Nailing without Bone Grafting for Humeral Shaft Nonunion Associated with Early Exploration of Secondary Radial Nerve Palsy: A Case Report

**DOI:** 10.3390/neurolint16050077

**Published:** 2024-09-15

**Authors:** Dan Viorel Nistor, Răzvan Marian Melinte, Romana von Mengershausen

**Affiliations:** Department of Orthopedics and Traumatology, Iuliu Hațieganu University of Medicine and Pharmacy, Strada Victor Babes 8, 4000132 Cluj-Napoca, Romania; dan88nistor@yahoo.com (D.V.N.); rmmelinte@yahoo.de (R.M.M.)

**Keywords:** humerus shaft, nonunion, intramedullary nail, radial nerve palsy, exploration, neurolysis

## Abstract

Background: Humeral shaft fractures are relatively common. Complications associated with this type of fracture and its treatment include nonunion and radial nerve palsy. Plate osteosynthesis with autologous bone grafting is considered the gold standard for treating nonunion. However, bone grafts might not always be necessary in cases of hypertrophic nonunion, and treatment should be tailored to the specific type and characteristics of the nonunion. The treatment of radial nerve palsy is debated, with some favoring expectant management based on the nerve’s ability to regenerate, and others preferring early surgical exploration to prevent possible lasting nerve damage. Methods: We present the case of a 46-year-old male patient with a six-year-old humeral shaft fracture resulting in hypertrophic nonunion. We treated the nonunion with anterograde intramedullary nailing without bone grafting. Postoperatively, the patient developed severe radial nerve palsy. After repeated electrophysiological studies, a decision was made to surgically explore the nerve 10 days after the nonunion surgery. The nerve was subsequently found to be intact and treated with neurolysis. Results: Bony union was shown at six months after nonunion surgery. Four months after the nonunion surgery, the patient started to show clinical signs of nerve recovery, and at 12 months he achieved nearly full clinical recovery of radial nerve function. Conclusions: Anterograde intramedullary nailing without autologous bone grafting may be considered an option for treating hypertrophic nonunion. The management of radial nerve palsy requires effective cooperation and communication between patient and physician. Further research is necessary to be able to better predict nerve recovery.

## 1. Introduction

Humeral shaft fractures (HSF) represent about 1–5% of all fractures [1,2] and 20% of all fractures of the humerus [3].

The choice of treatment stands in relation to the fracture type, displacement, and patient needs. Conservative treatment is considered by many to be the gold standard of treatment [1,3,4], as the outcomes are generally favorable with union rates between 77.4–100% [1,5]. Yet, in the past decades, the trend has shifted toward surgery [1,5,6,7] mostly with plate osteosynthesis or intramedullary nailing (IMN) [1,8]. Nonunion is a major complication of fracture treatment and can be defined as failure of the bone to unite within six months [9,10].

Nonunion of HSF occurs with conservative management as well as surgical treatment, even though the rate is significantly higher in conservatively managed fractures [2,3,8,10,11,12]. Moreover, it is associated with significant morbidity, poor function, and poor quality of life [2,13].

The classification of Weber and Cech (1976) is the most commonly used tool to describe the different types of nonunion: hypertrophic, oligotrophic, and atrophic. [14] Hypertrophic nonunion is biologically active and vital, while atrophic nonunion is associated with biological inactivity and lack of viability [2,9,15].

Treatment of nonunion is mostly performed surgically using a variety of techniques: plate fixation with or without autologous bone grafting (ABG), IMN with or without ABG, bone struts, or external fixation [2,16]. As a more advanced treatment strategy, synthetic bone graft substitutes have appeared in recent years and find increasing use [17].

A complication highly associated with HSF and its treatment, is radial nerve palsy (RNP), with a prevalence reported between 2–19% [1,7,18,19]. RNP can be classified into partial or complete, or into primary or secondary in accordance with time to appearance after injury. Primary injury appears at the time of injury, while secondary RNP occurs during treatment [20,21,22].

Seddon’s classification of peripheral nerve injury divides them into three categories, termed neuropraxia, axonotmesis, and neurotmesis. Another classification commonly used is Sunderland’s, which divides nerve injury into five degrees [23].

Neuropraxia, corresponding to Sunderland’s first degree, can be defined as nerve contusion with local nerve conduction block, with injury to the myelin sheath but intact axons. In axonotmesis, respectively, Sunderland 2, 3, and 4, injury to the axons has occurred while supporting structures (epineurium, endoneurium, and Schwann cells) remain intact to a variable extent. Neurotmesis (Sunderland 5) is a complete dissection of the nerve [24,25]. Some refer to a sixth Sunderland degree, representing a mixed nerve lesion involving both axonal damage and a conduction block [26].

Axonotmesis and neurotmesis are associated with Wallerian degeneration (WD). WD is a sequence of physiologic and metabolic changes characteristic of peripheral nerve injury (PNI) [27]. It is an innate immune-regulated mechanism initiated by axonal injury [28]. It starts 24–36 h post-injury and is completed within approximately 9–10 days, after which it can be objectively confirmed using electrophysiological studies (EPS) [25,26,29].

EPS, including nerve conduction studies (NCS) and electromyography (EMG), is used to localize and classify a nerve lesion according to type, severity, and prognosis, and for monitoring [26,29].

The management of RNP can be categorized under expectant treatment or early exploration. The former encompasses a watchful waiting attitude in accordance with the high rate of recovery of the radial nerve, without the need for surgical intervention [6,7,21,30]. If no signs of recovery appear within a period of 2–3 months, then late surgical exploration of the nerve can be considered [18,20,23]. Early exploration is performed within eight weeks from injury [7,18,23].

Depending on the intraoperative status of the explored nerve, neurosurgical treatment may consist of neurolysis, neurorrhaphy, nerve or tendon transfer [21,27].

The Quick Disabilities of the Arm, Shoulder, and Hand questionnaire (QuickDASH) is a widely used and well-tested instrument to assess upper extremity function. Lesser values indicate better function [31].

In the following, we present a case of a patient with longstanding hypertrophic, maligned nonunion of a humeral shaft fracture, treated with anterograde IMN, without ABG. Post-operatively, the patient developed severe RNP, which was managed with early surgical exploration and neurolysis.

## 2. Case Report

A 46-year-old, right-hand dominant male presented for evaluation of a non-united left HSF causing functional impairment affecting his daily activities and work performance. Six years earlier, he had suffered a transverse fracture of the middle third of his humeral shaft, which was treated surgically with plate fixation and seven screws in another service. Approximately two weeks postoperatively, the osteosynthesis material failed after repeated stress. The patient was treated in his hometown service, where it was decided to leave the material in place and continue the treatment conservatively with six weeks of immobilization in a U-slab cast. The patient was lost to follow-up until he presented to our service with the complaints described above.

On physical examination, the patient exhibited a varus deformity of the left brachium. Upon palpation, abnormal movement in the fracture site could be elicited. The patient’s main disturbance was functional impairment, particularly a lack of grip strength. Elbow and shoulder range of motion (ROM) were unaffected. 

**Figure 1:**
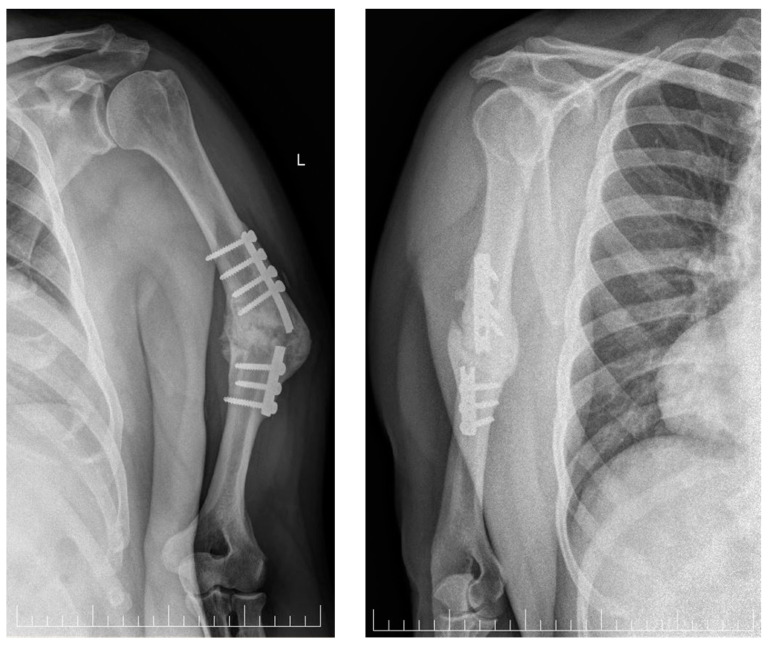
Pre-operative X-ray of the left humerus. Left: antero-posterior view, and right: latero-lateral view, showing hypertrophic nonunion of the humeral shaft fracture with the presence of deteriorated osteosynthesis material.

Plain radiographs showed a hypertrophic non-united humeral shaft fracture with 40° varus malignment and the presence of deteriorated osteosynthesis material, consisting of a plate and seven screws (Figure 1).

Surgical treatment was discussed with the patient, and he was informed about the risks of surgical correction. The patient chose to undergo operative treatment involving removal of the deteriorated hardware, resection of hypertrophic callus, and corrective osteotomy with fixation using anterograde IMN. His informed consent was obtained.

On the day of intervention, the patient received a preoperative interscalene nerve block under ultrasound guidance. The patient was placed in the beach chair position on the operating table.

The surgical approach was made laterally along the scar of the former incision. Through blunt dissection between the biceps and triceps, the humerus was exposed, revealing the presence of deteriorated osteosynthesis material partially included in the hypertrophic callus. 

Firstly, we removed the proximal part of the plate, during which the second screw broke, counting from superiorly. While removing the distal part, the most superior screw of the plate fragment broke. Using a hollow reamer, the remaining screw fragments were removed. 

The radial nerve was identified at the level of the distal humerus. The hypertrophic callus was exposed in a circular fashion, and corrective osteotomy was performed with resection of the callus. 

**Figure 2:**
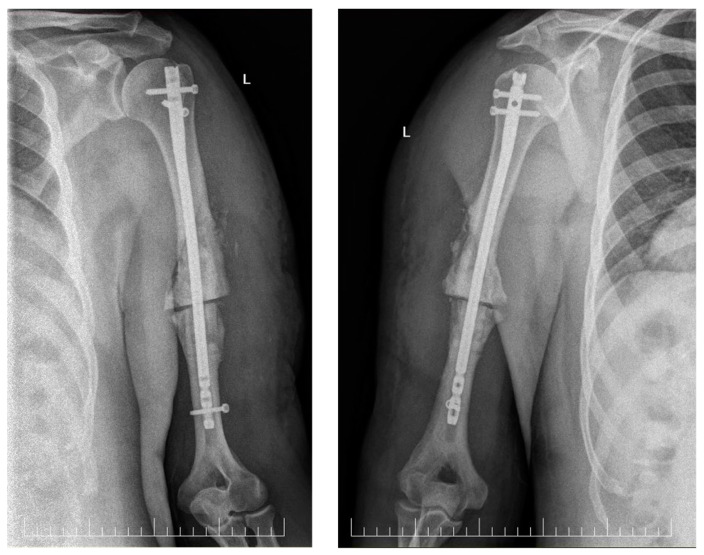
Postoperative X-ray of the left humerus. Left: antero-posterior view, and right: latero-lateral view, showing the IMN fixation.

We proceeded with the fixation of the osteotomy using an anterograde IMN. A 4 cm incision was made at the anterolateral level of the acromion, followed by dissection until exposure of the supraspinatus tendon. The tendon was incised in line with its fibers. Using an awl, the entry point for the nail was created and a guide wire was introduced under direct visualization of the osteotomy site. The humeral canal was prepared with 9 mm and 10 mm reamers. A 360/9 mm nail was selected and inserted. Proximal locking was performed percutaneously with two screws and distally under direct visualization, with the distal screw in dynamic mode. All screws were inserted in a transverse fashion (Figure 2). 

Placement was confirmed using intraoperative fluoroscopy. Shoulder and elbow ROM and stability of fixation were tested on the operating table. Layered wound closure was subsequently performed.

On the first postoperative day after cessation of the anesthesia effect, the patient continued to show a deficit of wrist and finger extension, and mild paresthesia in the territory supplied by the radial nerve. A neurologist was consulted and performed EPS on the third postoperative day which showed complete denervation of the target terrain of the radial nerve, excluding the long head of the triceps brachialis. According to the recommendation of the neurologist, the patient received dexamethasone 8 mg for seven days. A control EPS was undertaken on the seventh day, with the same result. 

**Figure 3:**
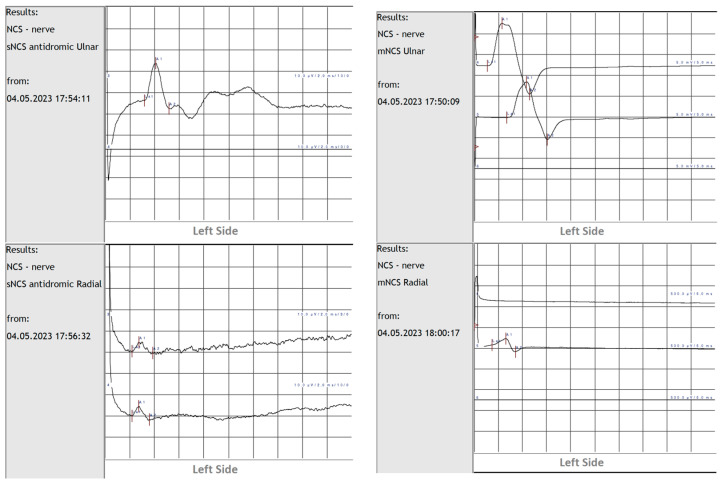
NCS on tenth postoperative day. Left: Sensory NCS (sNCS) left ulnar vs. left radial nerve, and right: motor NCS (mNCS) left ulnar vs. left radial nerve. Aspect of EPS on the tenth day after humeral nonunion surgery.

On the tenth postoperative day, the patient repeated the EPS with NCS and needle EMG (Figure 3), which showed an aspect of recent, severe neuropathy of the left radial nerve with a complete absence of motor response and signs of Wallerian degeneration. 

There was absent motor unit recruitment in almost all muscles supplied by the radial nerve, except the brachioradialis muscle which showed some fibrillations. The long head of the triceps brachii muscle had normal innervation. The location of the nerve lesion was therefore diagnosed to be inferior to the axilla, distal to the branch to the long head of the triceps. 

The performing neurologist’s recommendation was a surgical exploration of the radial nerve. This was discussed with the patient and with his consent he was transferred to the Department of Plastic Surgery.

The exploration was performed under general anesthesia on the 13th day after the nonunion correction. 

Intraoperatively, the plastic surgeon found that the radial nerve remained intact. They performed a decompression with partial excision of fibrotic tissue and evacuation of about 50 mL lysed hematoma, followed by neurolysis along the entire trajectory of the radial nerve in the brachium. At the level of the osteotomy site, the radial nerve was adherent to an approximately 0.5 mm bony spur from which it was liberated. 

Postoperatively, the patient reported improvement in sensory function of the radial nerve, without amelioration of the motor deficit.

The patient was transferred back to our service and discharged with instructions to begin physical therapy immediately. 

**Figure 4:**
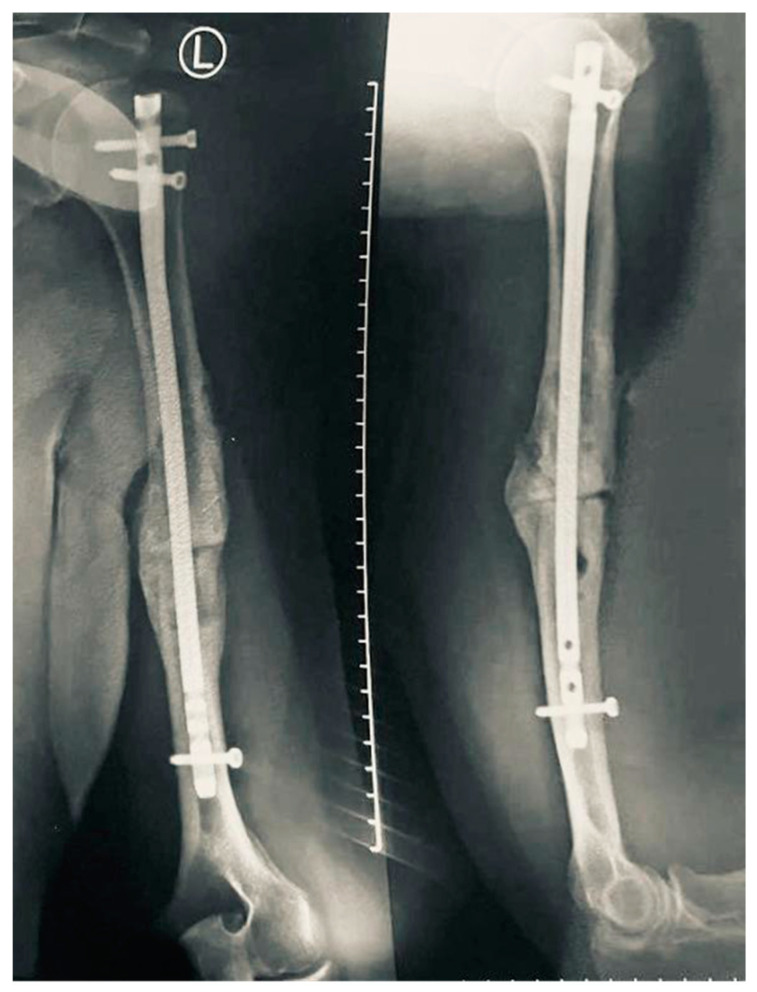
X-ray at six-month follow-up. Left: antero-posterior, and right: latero-lateral view, showing fracture consolidation in process.

Wound and fracture healing were uneventful. Six months after the nonunion surgery, bony union was confirmed via plain radiography (Figure 4). Four months after nonunion correction, the patient reported the first signs of radial nerve recovery. At 12 months, the patient reported a QuickDASH score of 11.4, with a score of 26 in the optional work module. Repeated EPS at 13 months post-neurolysis demonstrated aspects of chronic radial nerve neuropathy, with reduced amplitude and velocity in motor and sensory conduction of the left radial nerve, without signs of a conduction block or active denervation. 

## 3. Discussion

We described a case of longstanding hypertrophic, maligned nonunion of a humeral shaft fracture treated with anterograde IMN without ABG. Immediately after surgery, the patient developed an RNP with complete absence of motor response and partial sensory deficit. The RNP was treated with early surgical exploration and neurolysis. The onset of RNP recovery began at 16 weeks and almost fully recovered by 12 months.

The causes of impaired bone healing are diverse. Consequently, fracture nonunion is a heterogeneous entity, and its treatment should be tailored accordingly [9].

The requirements for successful fracture healing can be divided into biological and mechanical. Giannoudis et al. [32] introduced the “Diamond concept of fracture healing” consisting of three biological factors: osteogenic cell population, osteo-inductive stimuli, and osteoconductive matrix scaffold. The fourth factor is mechanical stability. 

In hypertrophic nonunion, the last factor is lacking, while the biology is intact [2,9,15,32,33,34]. Callus formation is the body’s physiological response to interfragmentary fracture mobility, attempting to reduce movement to be able to achieve consolidation. If a fracture treatment, surgical or conservative, is unable to keep the local strain <10%, no union can be expected as only fibrous tissue can support this amount of mobility. The result is a hypertrophic nonunion if there is adequate blood flow and residual cell vitality [15,32,33,34]. It is characterized by stiff or rigid mobility in the nonunion area [15].

Plate fixation with ABG is considered the gold standard treatment for nonunion [2,10,16,34]. Peters et al. [16] in their review of 36 studies aimed to compare union rates among operative strategies, and found union rates of 98% for plate fixation with ABG, compared to 95% without ABG. IMN with ABG had a union rate of 88% and without 66%. Bone struts had a union rate of 92% and external fixators 98%, but were associated with a higher rate of complications (20–22%). The type of nonunion was not taken into account. 

Oliver et al. [10] studied 86 patients undergoing ORIF with plate fixation with or without ABG and found no significant difference between the two cohorts. In addition, they found no significant difference between the treated nonunion types. Furthermore, 95% of nonunion united without supplementary ABG. 

Micic et al. [35] reported a 90% union rate in 20 patients with humeral shaft nonunion treated with locking IMN without ABG.

These high union rates without bone grafting have called the gold standard into doubt because the graft harvesting, with the anterior iliac crest being the most commonly used, is associated with considerate donor site complication rates of 20–39%, with infection, hematoma, fracture, pain and dysesthesias representing some of the potential complications [2,10].

It is advocated to take the type of nonunion and the underlying pathologic process into greater consideration when deciding on the nonunion treatment [33,34].

In our patient, we did not use ABG because the type of nonunion suggested a good local biological background for future healing, and the patient’s history of early failure of plate osteosynthesis after repeated stress led us to opt for IMN. 

IMN is less invasive, causes fewer circulatory problems, and has a lower risk of radial nerve injury than plate fixation [16,36]. It is able to provide stable fixation combined with load-sharing and allows for early weight-bearing and rehabilitation [33,37]. It was also found to have significantly lesser intraoperative blood loss, shorter operative time, and shorter hospitalization period when compared to plate fixation in the treatment of nonunion [35]. 

The reaming process associated with IMN also improves stability by enhancing the bone–nail contact area and increasing periosteal blood circulation, supporting bone formation. The reaming process carries important biological effects for nonunion healing, as its debris is rich in osteoprogenitor cells and growth factors, and it transports mesenchymal stem cells into the intramedullary space, which can be considered “internal bone grafting” [9,33].

The disadvantages of IMN include reduced intra-fragmentary compression compared to plate fixation, which in the humerus as a non-weight-bearing bone having intrinsically less axial compressive forces can lead to less consolidation [16]. Shoulder pain and stiffness are also more common with IMN [8,11,37].

Nonunion significantly impacts a patient’s health-related quality of life (QoL). Patients suffering from nonunion report lower QoL than most other patients in the musculoskeletal disorder population. It also ranks significantly lower than patients with chronic diseases such as chronic obstructive pulmonary disease, acute myocardial infarction, and stroke [13]. Even after successful achievement of the union, their QoL seems to remain below the standard reference population [38].

This does not change the importance of treating nonunion, as the effect on the patient’s situation is impactful, as suggested by Vincken et al. [13] when they seek to explain the large difference in QoL rates between their and another QoL study, theirs being much lower, but of a population of untreated nonunion patients, while in the other study patients had already reached boney consolidation [38].

Before any surgery, patients have to be thoroughly informed about the possible risks. Particularly associated with surgery of the humeral shaft are injuries to the radial nerve, due to its anatomical relation and variable course even under physiologic conditions [22,25,39].

The radial nerve starts as a branch of the posterior cord of the brachial plexus. It enters the posterior compartment of the brachium through the triangular fossa, crosses from medially to laterally during which it is in direct contact with the humeral periosteum for a distance of 6.3 cm ± 1.7 cm, and pierces the lateral intermuscular septum distally [40,41].

This close contact with the humerus and passage through rigid spaces, such as the intramuscular septum, leaves the radial nerve at increased risk for injury in cases of HSF and their treatment [18,20]. Iatrogenic RNP due to surgical management of HSF has an incidence between 1.9–3.3%, or even higher after non-union repair. A consecutive retrospective cohort review examined the rate of RNP in humeral shaft non-union surgery, finding 6.9% of 379 patients showed iatrogenic RNP. Among them, 15.8% had persistent deficits at twelve months follow-up [40,42].

The radial nerve displays a considerable capacity for self-regeneration, as 70–80% of primary RNP recover spontaneously, with an even higher rate if applied exclusively to secondary RNP [7,19,43,44]. The average onset of recovery is about 7–10 weeks and full recovery at 5–8 months [6,7,19]. Because of this, the management of RNP is a topic of debate, with the majority supporting expectant strategies with late exploration if indicated [1,7].

A generally proposed strategy is repeated controls using EPS at three-, six-, and twelve weeks [20]. Surgical exploration is recommended only in patients without signs of improvement after eight-to-twelve weeks [18,20,23]. Some support even longer observation periods, between four-to-five months and sometimes up to six months [19,43,45]. 

In 2005, Shao et al. [19] published a systematic review of RNP associated with HSF. They found no clinically significant difference in the final result between the early vs. late exploration group, suggesting that there was no negative effect upon nerve recovery when managed initially expectant. 

This can allow for a spontaneous return to function, avoiding unnecessary surgery [20,22,23]. By delaying surgery, the neurilemmal sheath has time to thicken, which might facilitate easier neurorrhaphy if late repair is indicated [19,20,22]. 

Ilyas et al. [7] performed an update of Shao’s systematic review. They included the cases of the previous study, thereby covering a time span from 1964 until August 2017. They divided the patients into initial expectant treatment, delayed surgical exploration (after eight weeks), and early surgical exploration. But unlike Shao et al. before, they did not combine expectant and late surgical treatment in the end, so as to compare the true outcomes of RNP treated early vs. late. The expectant group had a recovery rate of 77.2%. Palsies managed with early exploration within three weeks showed a significantly higher recovery rate of 89.9% (*p* < 0.001). Moreover, 68.1% of palsies undergoing late exploration recovered. This lower number was assumed to be associated with nerve retraction, distal motor end plate loss, muscular atrophy, and irreversibility of nerve injury due to delayed management. 

Ilyas et al. found this to challenge the dogma of expectant treatment. They concluded that the question must be asked whether a quota of 1:4 non-surgically treated patients with no spontaneous recovery is acceptable to withhold early surgical treatment.

Supporters of early exploration argue that delaying the intervention could compromise nerve recovery due to the risk of progression of nervous degeneration, which can lead to motor end plate loss and irreversible muscular atrophy [18,23,45] which develops after six months [44]. The decreased potential of motor neuron regeneration shows already after 7–8 weeks [18]. 

Early classification and characterization of nervous injury leads to early appropriate treatment, and faster and more complete/predictable recovery [18,23]. Reported rates of intraoperative radial nerve status vary. According to Ilyas et al. [7], the radial nerve was in continuity in 63.7%, incarcerated in 10.5%, and lacerated in 26.8%. Others found 95.9% to be in continuity, of which 10% were entrapped, and 14.1% to be transected [6]. Rasulic et al. [45] found 57.9% to be in discontinuity. Nerve incarceration and laceration bear poor prognosis for recovery [6,7].

Timing of surgery and surgical technique represent the only prognostic factors effectively influenceable by physicians, in contrast to the mechanism, type, and severity of injury, lesion site, and patient characteristics [45].

Early exploration means another intervention for the patient with the accompanying risks of infection, osteomyelitis, nerve devascularization, and interruption of the nerve’s natural environment [18]. Others consider this approach safer and easier than a delayed exploration in fibrotic and possibly anatomically distorted tissue [18,22,23].

Some consider iatrogenic RNP as an indication for early explorative management [18], while others opt for handling it the same expectant way as primary RNP because it was shown to have similar [19,43] or even better recovery rates [23].

Our patient underwent multiple EPS in the immediate postoperative period on the third-, seventh-, and tenth day, all showing absent motor recruitment distally to the long head of triceps brachii, locating the lesion inferior to the axilla. The recommendation for surgical exploration was given on the tenth postoperative day, after a week of treatment with high-dose corticosteroids showed no signs of improvement. 

NCS measures the velocity, amplitude, and latency of motor and sensory conduction by external stimulation. EMG records insertional activity, abnormal activity, and motor unit potentials (MUP) directly from the muscle, at rest and during contraction, using needle electrodes [26].

The limitations of EPS in the management of RNP rest in its time dependence. Before days 9–10 after injury, EPS is of no particular diagnostic value because it takes this time interval for WD to occur to differentiate between neuropraxia or a higher-grade injury. It takes three-to-four weeks for muscle fibrillations to develop, signifying axonal injury, which can be mixed injury with some axonotmesis, but also neurotmesis. For EPS to reach sufficient specificity and sensitivity to discern between axonotmesis and neurotmesis, four months have to pass [28,29,39,46].

The challenge lies in finding the balance between the time-dependent diagnostic tool and time-sensitive nerve injury, with the aim being to decrease the rate of avoidable surgery in self-limiting RNP and to increase the chance of timely reconstruction for severe lesions [46].

The EPS performed 13 months after neurolysis showed motor and sensory conduction velocity to be decreased and amplitude severely decreased. Yet, when considering functional outcomes, our patient reported a QuickDASH of 11.4, which can be considered recovered. In the optional work module, the patient’s score was 26, so his professional performance was not fully recovered at 12 months post-injury [31]. Şahin et al. [47] studied the correlation between electrophysiologic testing and clinical and functional outcomes in patients after traumatic PNI. They found no statistically significant association between EPS and functional recovery at a follow-up time of 11.6 months. Considering this discrepancy, the EPS should be interpreted carefully and in combination with the clinical and functional picture. 

Iatrogenic RNP can present a frustrating condition for both the patient and treating physician as there exists no gold standard of treatment and the possible causes for the injury are plentiful [29].

The exact cause for this particular RNP remains uncertain: compression by surgical instruments, hematoma formation, adherence to the bone—all or some or one or none of these could be the reason [45]. EPS located the lesion inferior to the axilla, distal to the radial nerve branch supplying the long head of the triceps brachii. This location appeared incongruently and distant from our surgical approach. One could explore the possibility that the long head of the triceps brachii received innervation from the axillary nerve rather than the radial nerve in up to 14% of cases [48].

In consultation with the anesthesiologist, the possibility was considered that the RNP could be a complication of the nerve block. PNI is an extremely rare complication of regional anesthesia with an incidence generally found to be ≤1% [49]. High injection pressure, neurotoxicity of the local anesthesia drug, or direct injury from the needle, can be causative for injury [49,50].

For nerve lesions treated with neurolysis, the remaining question is how the treatment affected the outcome, as the possibility exists that the nerve could have improved without the surgery [51].

When assessing the situation, it is important to consider the patient’s quality of life. Nerve injury with associated pain or paralysis can have a severe impact on their social and professional life. The inability to perform useful movements with the limb leads to underusage of said extremity, which may lead to joint stiffness, further prolonging rehabilitation and disability. Long-standing injuries are often accompanied by anxiety and depression and the treating physician needs to be aware of these psychological impacts. Effective communication and a strong physician–patient relationship are necessary throughout the process of recovery [29].

## 4. Conclusions

IMN without ABG may be considered a viable option for the surgical treatment of hypertrophic humeral shaft nonunion, as this type of nonunion has adequate biological potential for healing, rendering bone grafting superfluous in some cases. Further independent statistical analysis with a larger sample of patients and the application of reliability criteria is necessary. Before surgery, patients need to be counseled about the risk of developing post-operative RNP.

The decision between early surgical intervention or expectant management of RNP should be made in close cooperation between the patient and physician. It needs to be an informed decision by the patient, taking into consideration the patient’s needs, wishes, and general physical and mental health. The timing of radial nerve exploration remains controversial, with some advocating for early exploration to prevent irreversible nerve damage. Carefully designed studies are required to account for the numerous factors that could influence nerve recovery, such as patient factors, type of surgical fixation, timing and type of surgical exploration, and rehabilitation.

## Data Availability

The majority of underlying data are available as part of the article. Extended data not included in the article can be made available on request from the corresponding author.
